# The pressure relief protection effect of different strip widths, dip angles and pillar widths of an underside protective seam

**DOI:** 10.1371/journal.pone.0246199

**Published:** 2021-01-28

**Authors:** Shuhao Fang, Hongqing Zhu, Yujia Huo, Yilong Zhang, Haoran Wang, Feng Li, Xiaokuan Wang

**Affiliations:** 1 School of Emergency Management and Safety Engineering, China University of Mining and Technology-Beijing, Beijing, China; 2 State Key Laboratory of Coal Resources and Safe Mining, China University of Mining and Technology-Beijing, Beijing, China; Al Mansour University College-Baghdad-Iraq, IRAQ

## Abstract

To design underside protective seam strip layout. Similarity model experiments, numerical simulations and theoretical calculations are used to quantitatively study the pressure relief protection effect of different strip widths, dip angles and coal pillar widths of a thin underside protective seam under deeply buried conditions. The optimal strip width range is obtained according to the change law of strain during the mining process of the underside protective seam in a similar model experiment. The change law of the expansion of the protected coal seam is obtained and the fitting surfaces among the dip angle and strip width of the coal seam with the protection distance and pressure relief angle along the strike and dip of the protected coal seam are established according to the numerical simulation results of underside protective seam mining. It is concluded that the best pressure relief effect can be achieved when the dip angle is 16.7° and the strip width is 70 m. According to the stability threshold of coal pillars considered in strip mining theory, the coal pillar width is calculated to be 50 m. Similarity model experiments and numerical simulations of protected coal seam mining verify the pressure relief effect of the designed protective seam strip width and pillar width. A calculation method of the protective seam strip width, position and pillar width required by the specific width of the protected seam is proposed.

## Introduction

The mine studied here is a coal and gas outburst mine, with a thin, deeply buried #4 coal seam, a complex geological structure including many faults, and poor economic benefits. Therefore, strip mining of the #4 coal seam as an underside protective layer not only solves the problem of the difficult mining of the #4 coal seam but also protects the #2 coal seam to a certain.

Because coal is an important energy source [1–8], research on the safety of coal production has become more detailed [9–15]. The development law of mining fractures of overlying strata and the traditional “three zones” theory is the basis for the research of first mining the underside coal seam mining to protect the coal group [16–20]. The key stratum theory is the mainstream theory for studying overlying rock layers [21–26]. Liang et al. [27] demonstrated that the first subordinate key stratum has six types of movement. Gao et al. [28] showed that the first fracture occurrence of the key stratum increases the displacement and stress and that the second fracture partially releases the stress. Sampath et al. [29] and Xie and Xu [30] studied the abutment pressure during coal mining based on the key stratum theory.

Similarity model experiments and numerical simulations are common methods of studying the “three zones” failure mode and related fractures in strata overlying shallow thick coal seams [31–35]. Ghabraie et al. [36] concluded panel configurations of two seams through various sand-plaster similarity model experiments, which have a significant impact on multiseam subsidence development. Wang et al. [37] obtained the height of an air conducting fracture zone through similarity model experiments and numerical simulation. He et al. [38] concluded that the movement boundaries of bedrock and unconsolidated strata are located away from the coal mining boundary based on the method of similarity model experiments and numerical simulation. Kang et al. [39] used similarity model experiments to research roof collapse during longwall coal retreat mining. Le et al. [40] studied longwall top coal caving behavior through discontinuous modeling. Yang et al. [41] studied the failure law of the overlying rock layer of a coal seam and the height of the "three zones" based on similarity model experiments. In three-dimensional similarity model experiments, the deformation law of overburden caused by continuous coal seam mining was studied [42]. Pan et al. [43] studied the caving interval of the hard roof during the mining of the lower coal seam based on similarity model experiments under conditions of premining and non-premining the upper coal seam. Zhou et al. [44] concluded that fracturing undergoes two development cycles (and two peaks) based on the method of theoretical analysis and similarity model experiments.

Protective seam mining in outburst mines is an effective means of eliminating the outburst risk of the coal seam. Li et al. [45] used a numerical simulation method to study the surface settlement during strip mining with different coal pillar widths. Through similarity model experiments and numerical simulations, Gao et al. [46] studied the change law of overburden stress after protective layer mining. Dong et al. [47] studied coal and gas outburst control by protective coal seam mining based on numerical simulation and related theories. Zhang et al. [48] studied the stress zoning of the upper and underside protected coal seam after protective seam mining through FLAC3D numerical simulation. Tu et al. [49] studied the stress evolution and deformation of the protected coal seam caused by remote upper protective seam mining based on FLAC3D numerical simulation. Jia et al. [50] studied the permeability distribution of the protected coal seam caused by protective coal seam mining through numerical simulation. Fang et al. [51] studied the pressure relief protection effect of upper protective seam mining with different coal seam dips through similarity model experiments. Zhang [52] studied the distribution law of floor stress during upper protective seam mining through theoretical calculations and numerical simulations.

Compared with experiments, numerical simulation research has the advantages of saving time and effort. The research adopts multiple numerical simulations and one experimental comparison to ensure the efficiency and accuracy of the research. The quantitative relationship between the dip angle, strip width and pillar width of the #4 thin coal seam that is deeply buried and the protection distance and pressure relief angle of the strike and dip of the protected #2 coal seam is established. The research has reference significance for the engineering design of the protective seam strip layout.

## Similarity model experiment

The average thickness of the main #2 coal seam is 5 m. The average thickness of the #4 coal seam is 1.3 m, the consistent coefficient of the coal seam is 0.54, and the average burial depth is 600 m. There are two surface boreholes in the mining face, and the lithology and thickness of the overburden rock at the working face can be obtained by the comprehensive stratigraphic column of the mine. The overburden rock parameters of the mining face are shown in Table 1.

**Table 1:** Overburden parameters.

No.	Rock stratum	*H*_*i*_/m	*h*_*i*_/m	*P*_*ai*_/MPa	*P*_*bi*_/MPa	No. ratio
19	Siltstone	120	26	90	0.56	337
18	Fine sandstone	94	10	110	0.69	328
17	Medium sandstone	84	10	130	0.81	319
16	Siltstone	74	7	90	0.56	337
15	Mudstone	67	3	40	0.25	519
14	Medium sandstone	64	11	130	0.81	319
13	Siltstone	53	8	90	0.56	337
12	#2 coal	45	5	10	0.06	773
11	Fine sandstone	40	4	110	0.69	328
10	Siltstone	36	6	90	0.56	337
9	Siltstone	30	7	90	0.56	337
8	Sandy mudstone	23	2	50	0.31	428
7	Fine sandstone	21	1	110	0.69	328
6	Siltstone	20	4.5	90	0.56	337
5	#3 coal	15.5	0.5	10	0.06	773
4	Siltstone	15	4	90	0.56	337
3	Fine sandstone	11	1	110	0.69	328
2	Sandy mudstone	10	2	50	0.31	428
1	Limestone	8	3	70	0.44	419
0	#4 coal	5	1.3	10	0.06	773
-1	Fine sandstone	3.7	3.7	110	0.69	328

Note: *H*_*i*_—total thickness at the i-th layer; *h*_*i*_—thickness of layer *i*; *P*_*ai*_—prototype compressive strength of layer *i*; *P*_*bi*_—model compressive strength of layer *i*.

According to the theory of mine pressure control [53–55], the maximum heights of the caving zone and the fractured zone are calculated by Eq (1) and Eq (2) when the overburden is hard [56, 57].

$$H_{c}=\frac{100M}{2.1M+16}\pm 2.5$$ (1)

$$H_{f}=\frac{100M}{1.2M+2}\pm 8.9$$ (2)

where *H*_*c*_ is the height of the caving zone, m; *M* is the mining height of the coal seam, 5 m, and *H*_*f*_ is the height of the fractured zone, m.

The coal seam #2 overburden is mainly composed of hard rock, therefore, the height of the caving zone and the fractured zones are 16.37~21.37 m and 53.6~71.4 m, respectively. The calculation here does not consider protective seam mining.

### Model establishment

The similarity model experiment is based on the two-dimensional test bed of the laboratory of China University of Mining and Technology (Beijing). The length, width and height of the test bed are 1800 mm, 160 mm and 1200 mm, respectively. According to the similarity principle [58], the geometric similarity ratio between the entity and the model is set as 100, the time similarity ratio is set as 10, the gravity similarity ratio is set as 1.6, and the stress similarity ratio is set as 160. The compressive strength of each layer is shown in Table 1. The No. ratio of similar materials corresponding to the compressive strength of rocks can be obtained according to the similarity ratio [59], as shown in Table 1.

**Fig 1:**
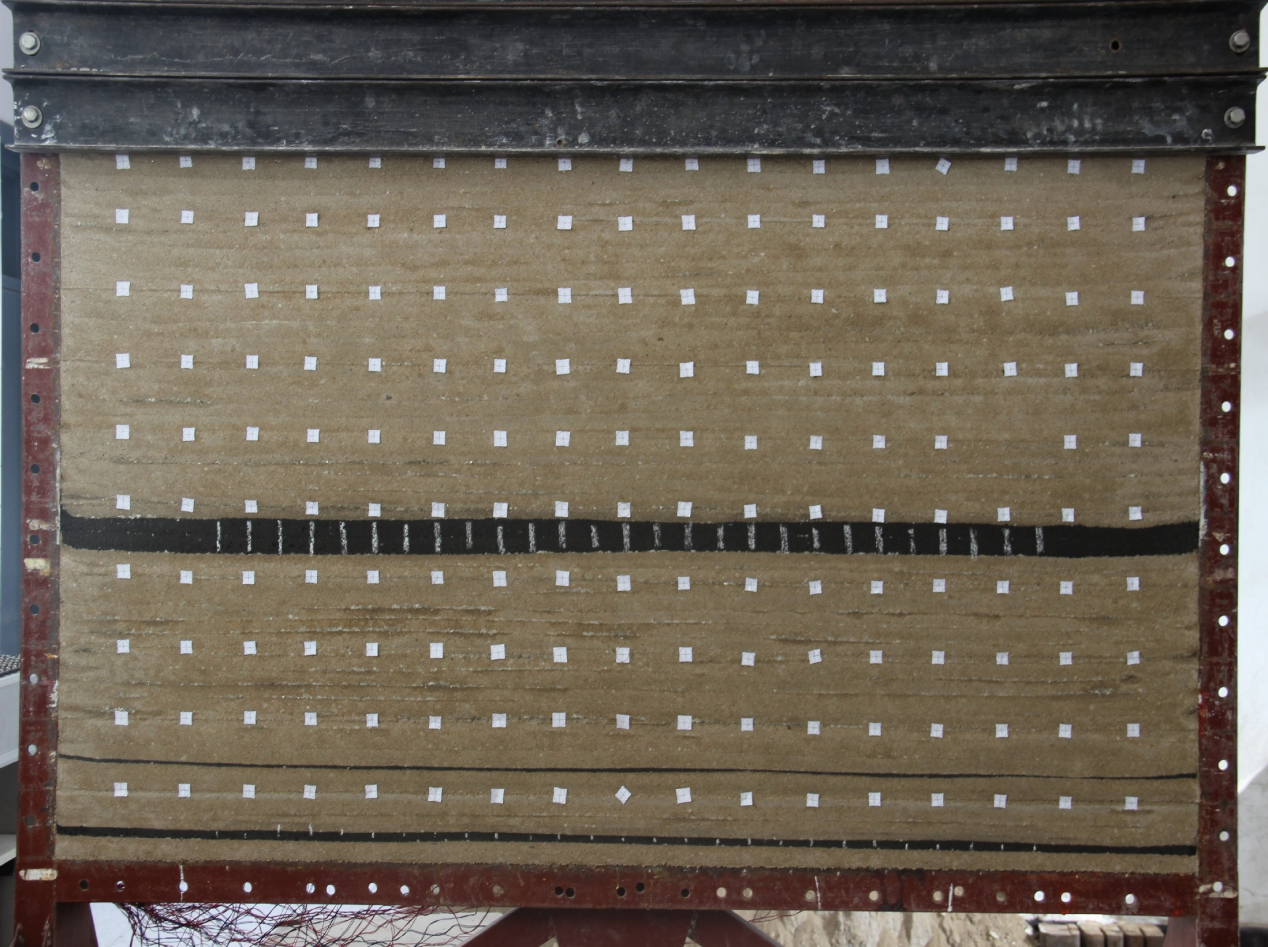
Experimental model.

The similar materials were sand, lime, gypsum, and water, and the model materials were proportioned according to the similarity ratio. Mica slices were placed between adjacent layers to simulate stratification. The physical model was constructed layer by layer with a thickness of approximately 10 mm and a total thickness of 1.2 m. The remainder of the height is only simulated to the surface with a counterweight. The completed physical model was allowed to dry naturally. The physical model after removing the mold is shown in Fig 1.

In the model, the resistance strain gauges were arranged 10 cm above the #2 and #4 coal seam roofs, and a total of 34 strain observation points were arranged at a horizontal interval of 10 cm. The resistance strain gauges buried in the experimental model were connected to a laptop through a DH3816 static strain test system, and the strain results were displayed on the laptop through supporting software.

### #4 coal mining

**Fig 2:**
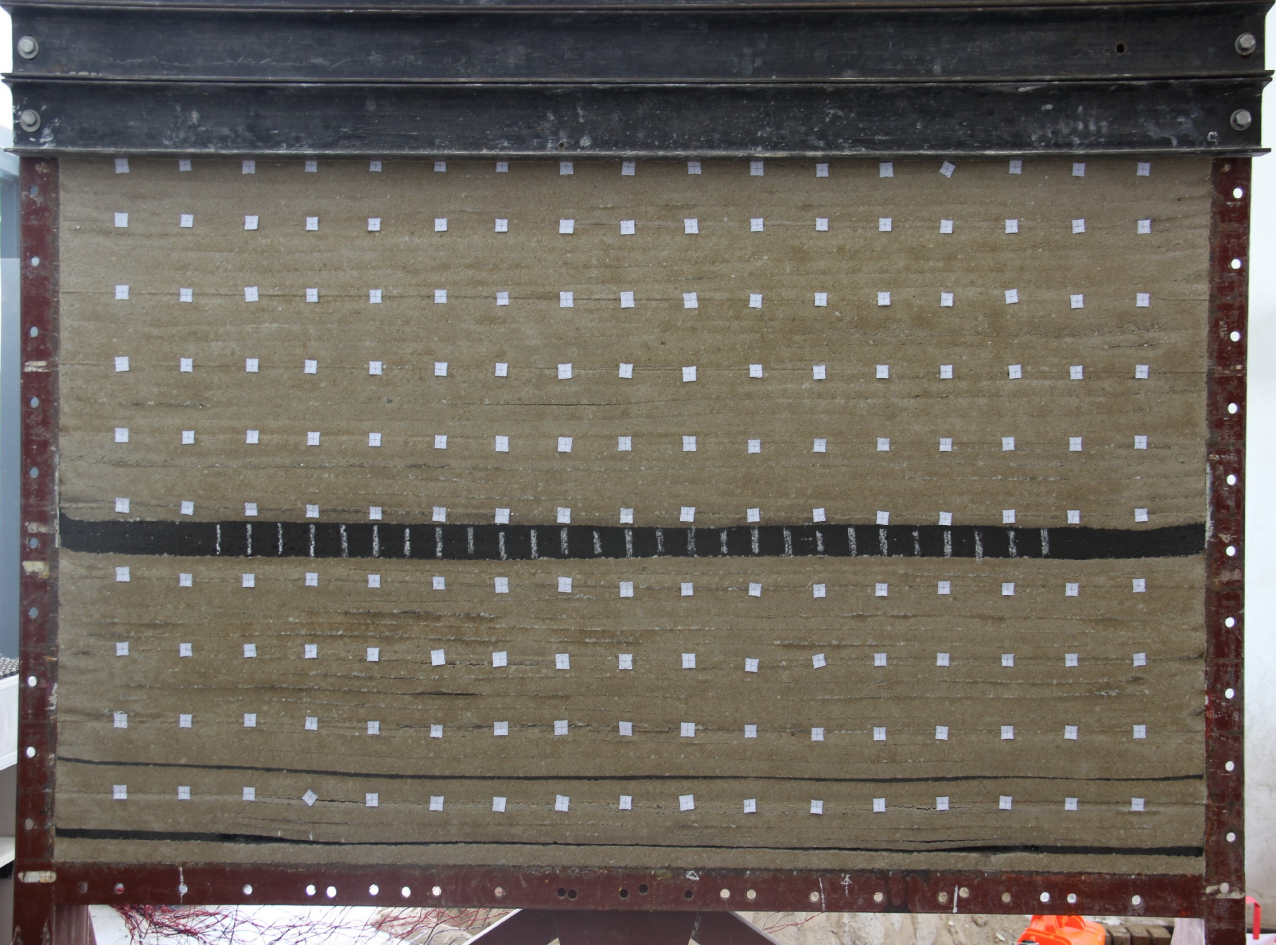
Overburden status at mining 130 cm.

In the model, the displacement, strain, fracture angle, interval of roof collapse, and height of roof collapse development were recorded for each 5 cm of mining. The distance between both ends of coal seam #4 and the boundary is 25 cm. The overburden status at the coal mining depth of 130 cm corresponding to coal seam #4 is shown in Fig 2.

Because coal seam #4 is relatively thin, roof fracture of the coal seam is not obvious during mining. With the mining of coal seam #4, the caving zone supports the overlying rock mass, the coal roof collapses periodically, and the fracture angle of the overburden at the mining side changes periodically. The mean fracture angle at the mining side is less than that of the open-off cut side, and the fracture angle at the side of the open-off cut is approximately 60°. When the coal seam #4 is mined to a depth of 75 cm, the coal seam #2 exhibits obvious separation cracks, and when the coal seam #4 is mined to a depth of 130 cm, the coal seam #2 curve subsides, indicating that the coal seam #4 has a depressurization effect on the coal seam #2.

**Fig 3:**
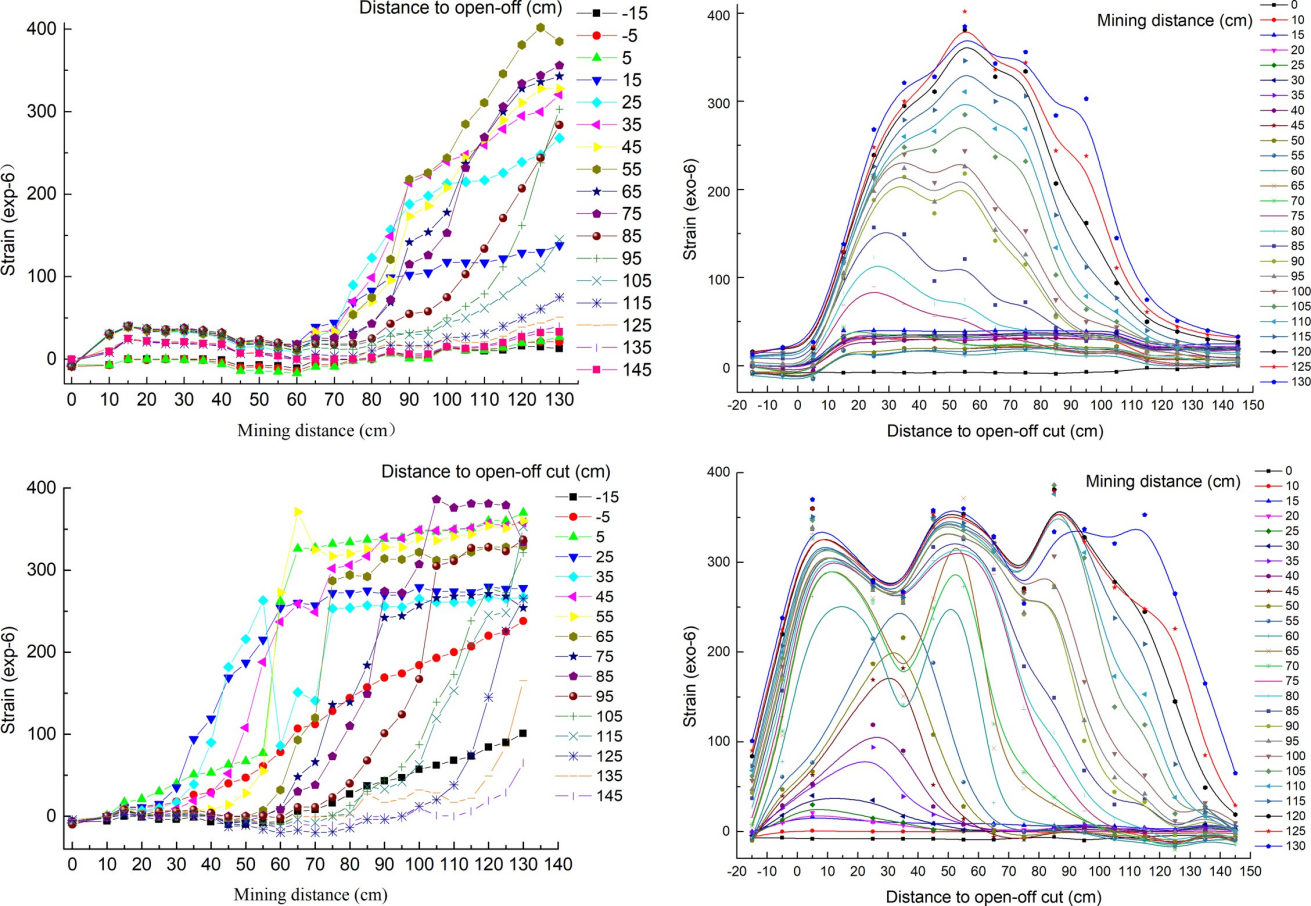
Relationship between the strain and mining distance. (A) Strain at points above coal seam #4. (B) Distribution of strain at points above coal seam #4. (C) Strain at points above coal seam #2. (D) Distribution of strain at points above coal seam #2.

Every 5 cm of mining, before continuing, the value at every strain point was measured and recorded. The change in strain value at each point with mining distance and the strain distribution at each point at different mining distances are shown in Fig 3.

After mining, the strain at each strain monitoring point increased slightly, then increased rapidly, and finally tended to be stable. Due to the periodic fall of the roof of the coal seam #4, there is a small periodic fluctuation in the strain above the coal seam #4. The strain value above the coal seam #2 is less affected by periodic collapse.

When the coal seam #4 is at a mining depth of 30 cm, the strain at 10 cm above the coal seam #4 begins to change significantly; when the mining depth ranges from 30 cm to 45 cm, the maximum strain change is observed; when the mining depth is greater than 55 cm, the rate of increase in the strain is very low. It can be concluded that when the shortest strip width of the coal seam #4 is greater than 55 cm, the pressure relief effect of the coal seam #4 can be achieved.

When the coal seam #4 is at a mining depth of 70 cm, the most obvious change in strain occurs at 10 cm above the coal seam #2; when the mining depth range from 70 cm to 90 cm, the strain change is the largest; when the mining depth is greater than 90 cm, the rate of increase in the strain is very low. The shortest strip width of the coal seam #4 that relieves the pressure of the coal seam #2 is less than 90 cm.

The similarity model experiment of coal seam #4 mining shows that the optimal range of the strip width of the coal seam #4 is 55~90 m due to the model similarity ratio of 100.

### Coal seam #2 mining

**Fig 4:**
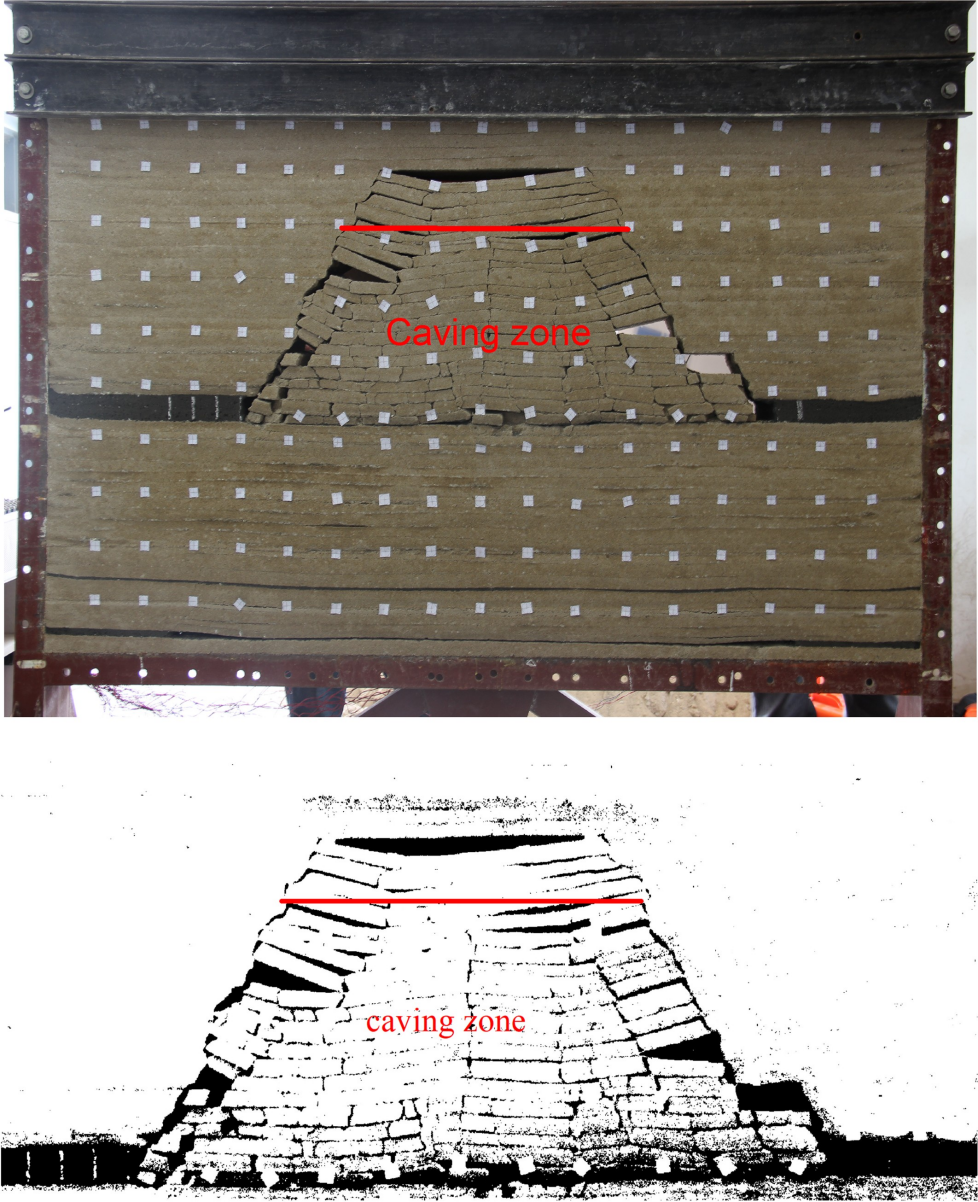
Overburden status of the coal seam #2 at a mining distance of 110 cm. (A) Overburden status. (B) Overburden status after binarization.

The distances between the open-off cut side and the mining side of coal seam #2 and the boundary are 40 cm and 30 cm, respectively. The overburden status of the coal seam #2 at a mining distance of 110 cm is shown in Fig 4.

After the coal seam #4 is mined, during the coal seam #2 mining, the interval of roof breaking is small, and the cracks in the overlying strata are fully developed. The height of the caving zone of the coal seam #2 is approximately 30 m due to the model similarity ratio of 100, which is greater than the theoretical calculation value. It is concluded that the coal seam #4 protective layer provides a pressure relief effect.

## Pressure relief protection effect of different strip models

### Numerical model

Based on FLAC3D numerical simulation software, a mechanical model of the coal mining face is established. The model mainly studies the pressure relief protection effect during strip protective seam mining, and the *Mohr Coulomb* constitutive model is used to establish this mechanical model. According to the geological conditions of the working face, the dip (y-axis direction), strike (x-axis direction) and height (z-axis direction) of the model are 200 m, 500 m and 250 m, respectively. According to the burial depth of the coal seam and the gravity of the overburden, the vertical stress is 15 MPa and the horizontal stress is 12 MPa. The monitoring points are set to monitor the changes in the stress and displacement of the overlying strata during mining. The physical and mechanical parameters of the strata in the model are shown in Table 2.

**Table 2:** Model parameters.

Lithology	Cohesion (MPa)	Shear modulus (GPa)	Bulk modulus (GPa)	Density (kg/m^3^)	Tensile strength (MPa)	friction angle (°)
Siltstone	2.3	1.009	2.02	2590	0.86	33
Fine sandstone	2.6	1.522	2.914	2670	0.93	34
Medium sandstone	3.2	1.84	3.843	2610	0.97	38
Limestone	4.1	2.6	5.2	2650	1.08	40
Mudstone	3.51	1.26	1.612	2400	0.8	34
Sandy mudstone	2.1	1.26	1.612	2500	0.6	36
Coal	2	0.368	1.189	1400	0.03	34

A preliminary grid model is established and can be solved to generate the initial ground stress field. After the superimposed force is applied to the model, the use of empty cell excavation is used to simulate the coal seam mining face. The model is divided into 26 adjacent layers with different lithologies. The strike of the strip is mined from x = 100 m to x = 400 m. Each time the model unit is mined 20 m, the unbalanced force ratio is set to 1e-5, and 15 excavation steps are used.

**Fig 5:**
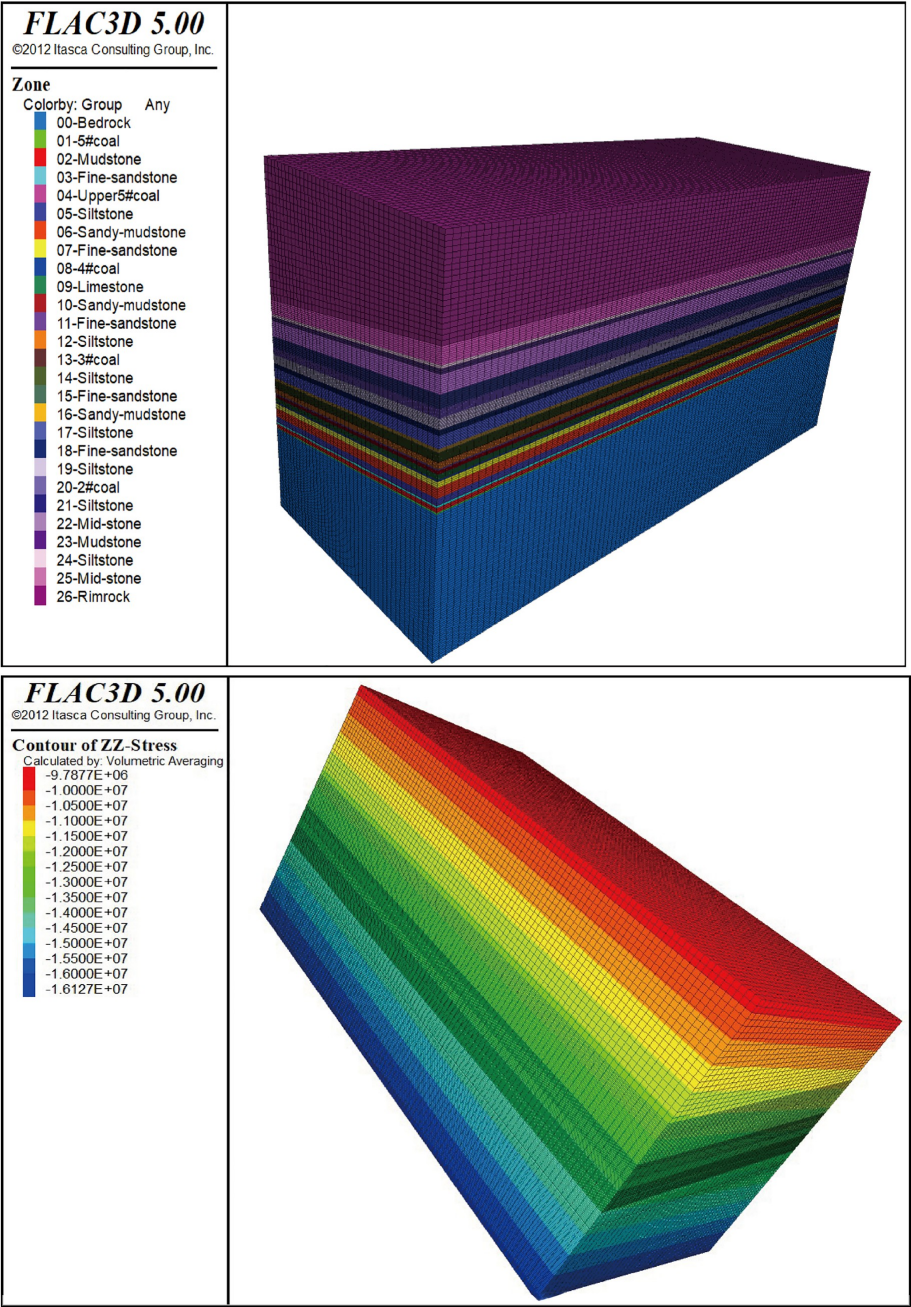
Numerical model when the dip angle is 10°. (A) Numerical model status. (B) Initial balance state of the vertical stress.

Models with the same parameters and different dip angles are established, and different strip widths are mined from the coal seam #4 in each model. When the dip angle is 30°, the horizontal strip widths are 30 m, 40 m, 50 m, 60 m, 70 m, and 80 m; when the dip angle is 20°, the horizontal strip widths are 35 m, 45 m, 55 m, 65 m, 75 m, and 85 m; when the dip angle is 10°, the horizontal strip widths are 40 m, 50 m, 60 m, 70 m, 80 m, 90 m; when the dip angle is 0°, the strip widths are 40 m, 50 m, 60 m, 70 m, 80 m, and 90 m; thus, there are a total of 24 mining models. When the coal seam dip is 10°, the numerical model state and the initial balance state of the vertical stress are shown in Fig 5.

### The dip protection effect produced by the dip angle and strip width

**Fig 6:**
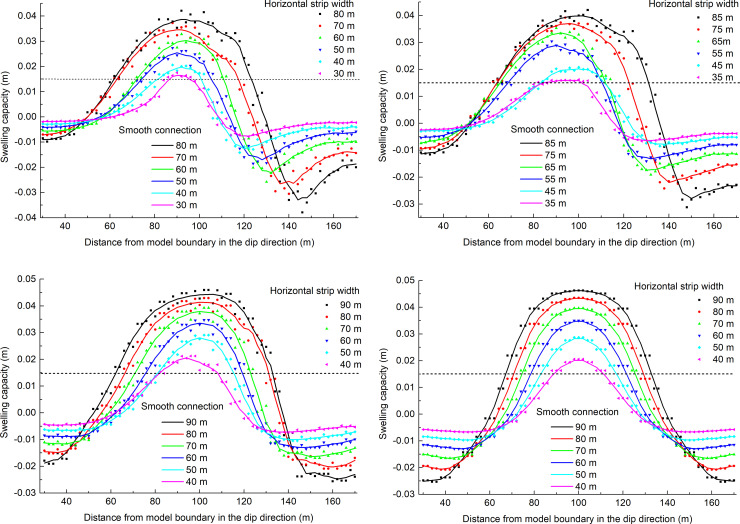
Distribution of swelling capacity of coal seam #2 in the dip direction. (A) The dip angle is 30°. (B) The dip angle is 20°. (C) The dip angle is 10°. (D) The dip angle is 0°.

When studying the pressure relief protection effect of the underlying coal seam mining on the overlying coal seam, the expansion rate of the protected seam is an important index for evaluating the protection effect. The increase in the expansion rate has a great impact on enhancing the permeability of the overlying coal and rock seam. According to the "Detailed rules for prevention of coal and gas outburst", the maximum expansion rate of the protected layer is 3‰ and serves as a critical index at which to measure the protective effect of the protective layer. The average thickness of the coal seam #2 is 5 m, and the swelling capacity of 3‰ is 0.015 m. To quantitatively analyze the pressure relief effect of coal seam #4 mining on the coal seam #2, 71 displacement monitoring points are arranged at equal intervals on the #2 coal roof and floor along the dip. Based on the displacement of coal seam #2, when the dip angles are 30°, 20°, 10°, and 0°, the swelling capacity distributions of coal seam #2 in the dip are shown in Fig 6 for different strip widths.

When the dip angle is the same, the swelling capacity of the coal seam #2 increases with increasing strip width, and the rate of increase in the speed decreases; in the dip direction, both ends of the coal seam #2 are in a compressed state, and the lower part of the coal seam #2 undergoes a larger compression. It could be concluded that the larger the strip width is, the better the pressure relief effect.

**Fig 7:**
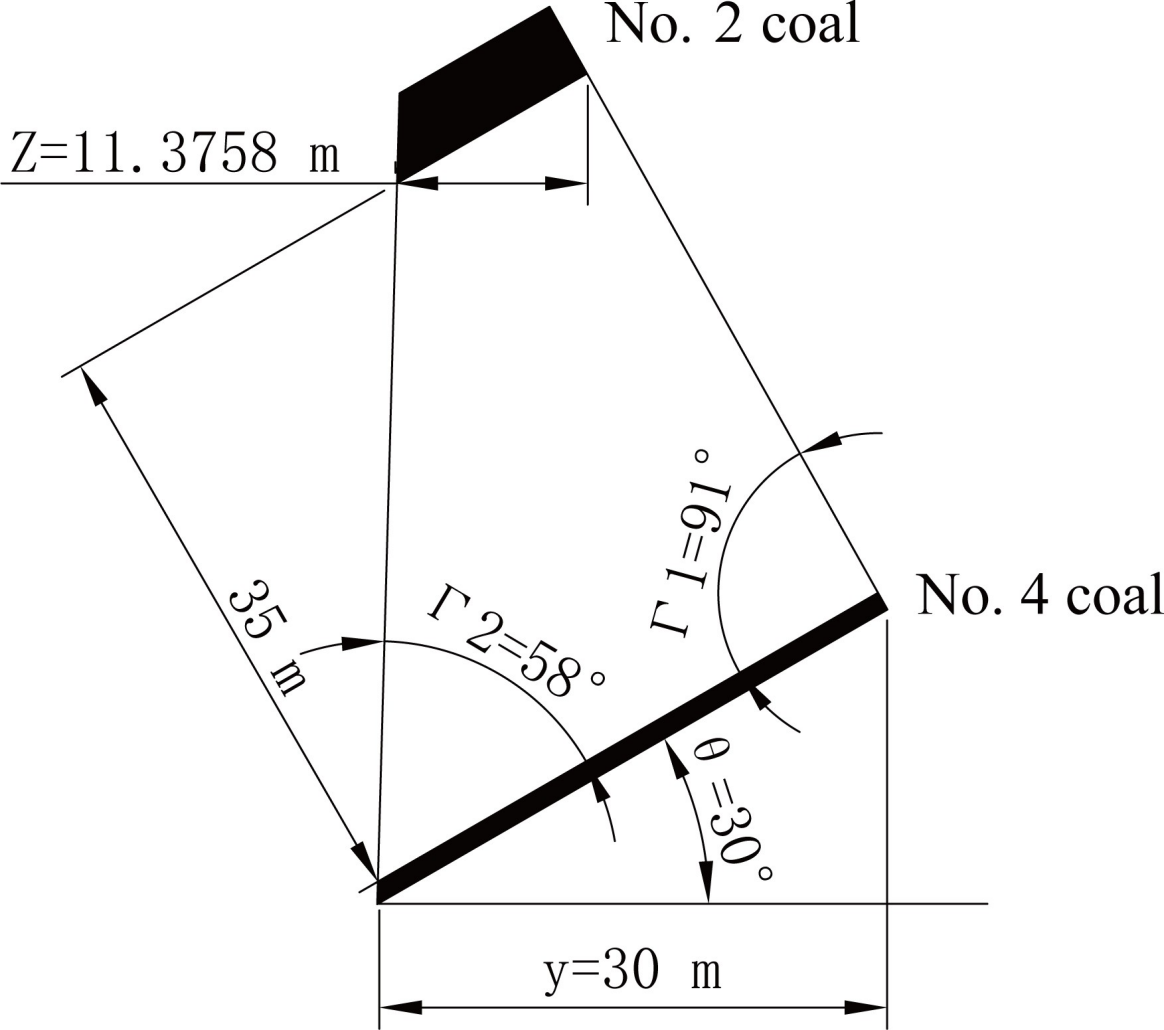
Schematic diagram of the pressure relief protection effect. Note: *θ*—dip angle, °; *y*—horizontal strip width, m; *Z*—horizontal pressure relief protection width, m; *Γ*_1_—pressure relief angle in the upper part, °; *Γ*_2_—pressure relief angle in the lower part, °.

**Fig 8:**
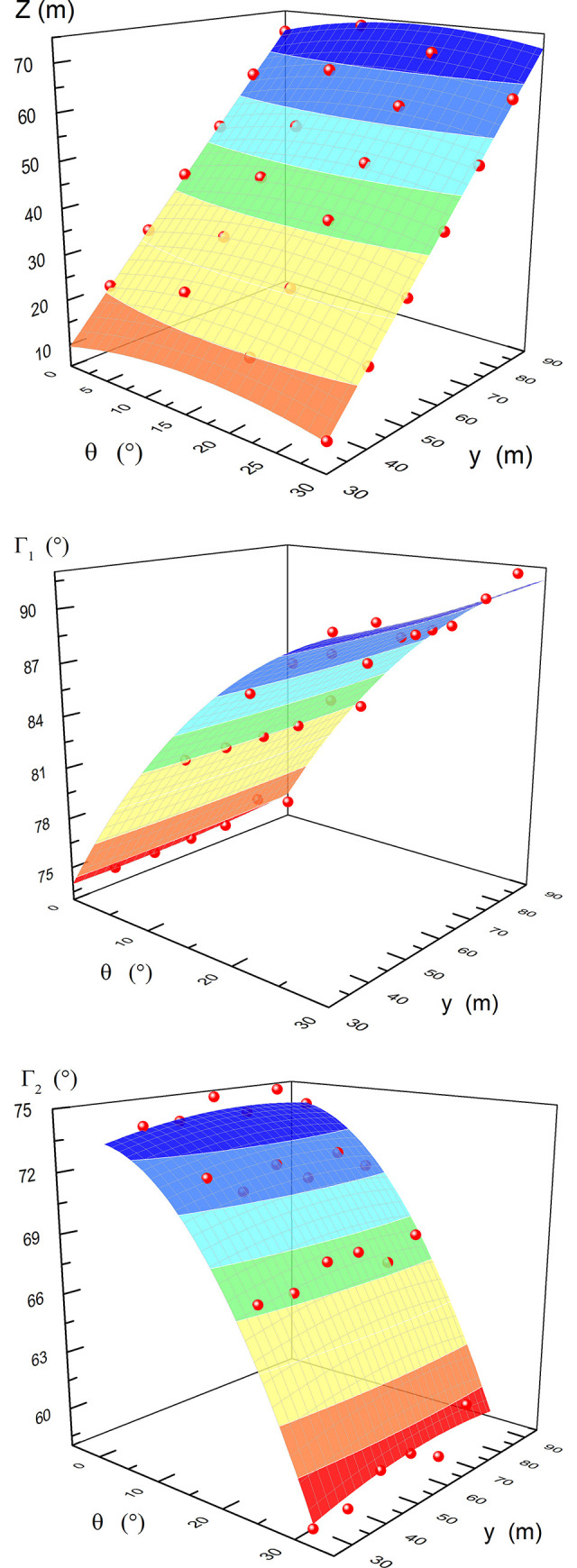
Fitted surfaces. Note: the red balls are the actual values. (A) Horizontal pressure relief protection width. (B) Pressure relief angle in the upper part. (C) Pressure relief angle in the lower part.

According to the distribution of the swelling capacity of coal seam #2 in the dip direction, the pressure relief protection parameters under the 24 sets of model test conditions can be obtained (as shown by the red balls in Fig 8). When the dip angle is 30° and the horizontal strip width is 30 m, a schematic diagram of the pressure relief protection effect is shown in Fig 7.

*θ* and *y* are independent variables, and *Z*, *Γ*_1_ and *Γ*_2_ are the dependent variables. Using Origin, the Parabola2D fit is selected for the fitting process; the fitting equations are shown in Eqs (3), (4) and (5), respectively, and the fitting diagrams are shown in Fig 8.

$$Z=-21.6191+0.5116\theta +1.0464y-0.015{3\theta }^{2}-0.0003y^{2}\;R^{2}=0.9990$$ (3)

$$\Gamma_{1}=74.4003+0.9178\theta -0.0222y-0.0120\theta^{2}-0.0003y^{2}\;R^{2}=0.9939$$ (4)

$$\Gamma_{2}=71.4673-0.0616\theta -0.0833y-0.0148\theta^{2}-0.0006y^{2}\;R^{2}=0.9851$$ (5)

In Eqs (3), (4) and (5), *θ* ranges from 0~30, *y* is ranges from 30~90, and the correlation coefficient (*R*^*2*^) is greater than 0.98.

*Z* is affected more by *y* than by *θ*, and *Z* has an approximately linear relationship with *y*. *Z* is less affected by *θ*, and *Z* reaches its maximum value at *θ* = 16.7°; Z has a parabolic function relationship with the *θ*. *Γ*_1_ is mainly affected by *θ*, *Γ*_1_ increases with increasing *θ*, and the rate of increase gradually decreases. The change in *Γ*_2_ is relatively small and is mainly affected by *θ*. *Γ*_2_ decreases with increasing *θ*, and the rate of decrease gradually increases. It is concluded that the dip angle of the coal seam with the best pressure relief effect is 16.7°.

Under the condition of a known coal seam dip, according to fitting Eq (3), the strip width required by the seam pressure relief protection in the dip direction can be obtained. From the obtained strip width, the pressure relief angle can be obtained according to fitting Eqs (4) and (5), that is, the strip position corresponding to the protected seam can be inverted in the dip direction.

### The strike protection effect produced by the dip angle and strip width

**Fig 9:**
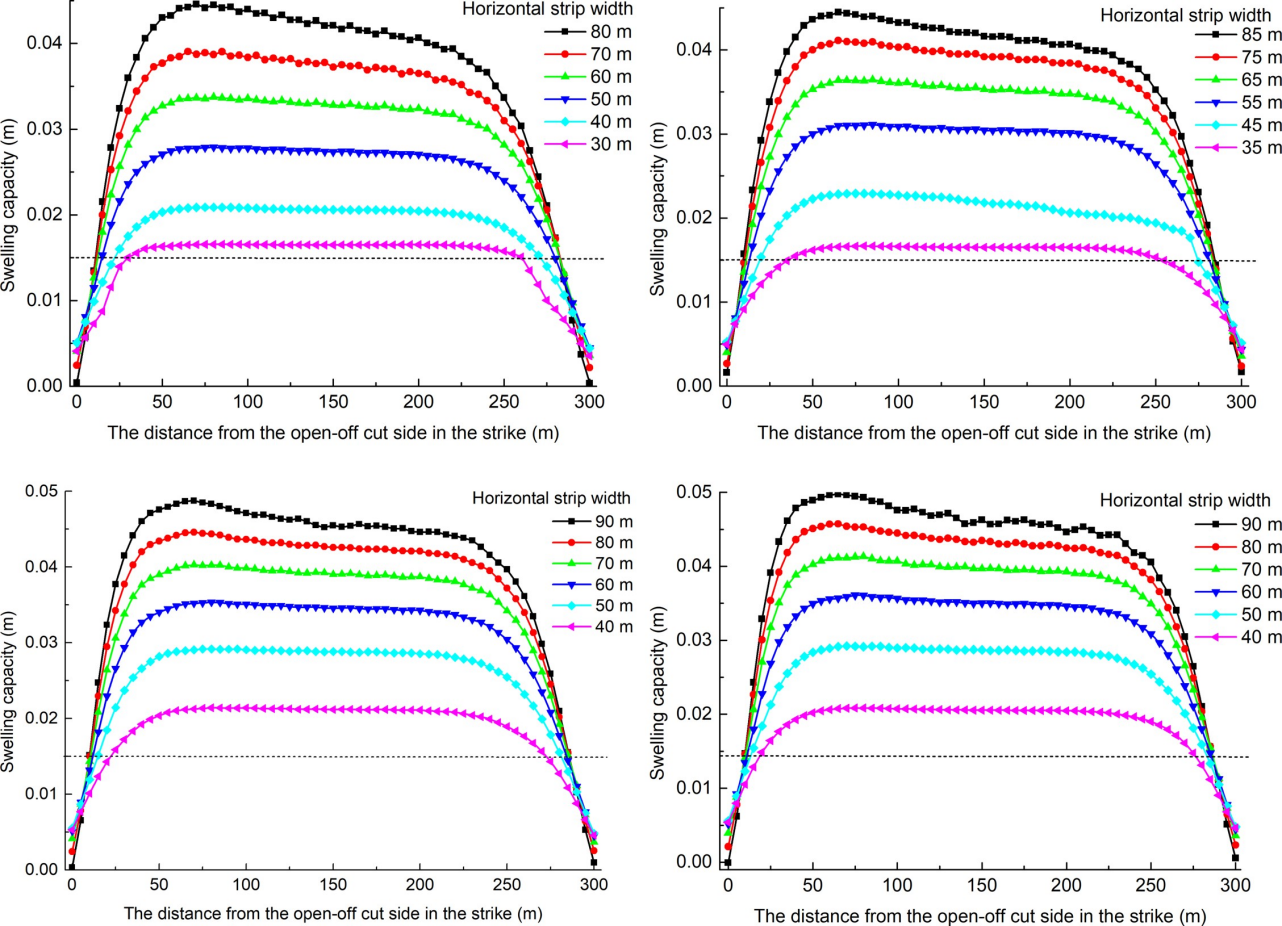
Distribution of swelling capacity of coal seam #2 in the strike direction. (A) The dip angle is 30°. (B) The dip angle is 20°. (C) The dip angle is 10°. (D) The dip angle is 0°.

Seventy-two displacement monitoring points are arranged equidistantly on the #2 coal roof and floor in the strike direction. Based on the displacement of coal seam #2, when the dip angles are 30, 20°, 10°, and 0°, the swelling capacity distributions for the different strip widths of coal seam #2 in the strike direction are shown in Fig 9.

When the dip angle is the same, the swelling capacity of coal seam #2 increases with increasing strip width, and the rate of increase in the speed decreases; in the strike direction, the expansion of coal seam #2 at the open-off cut side is greater than that at the mining side.

**Fig 10:**
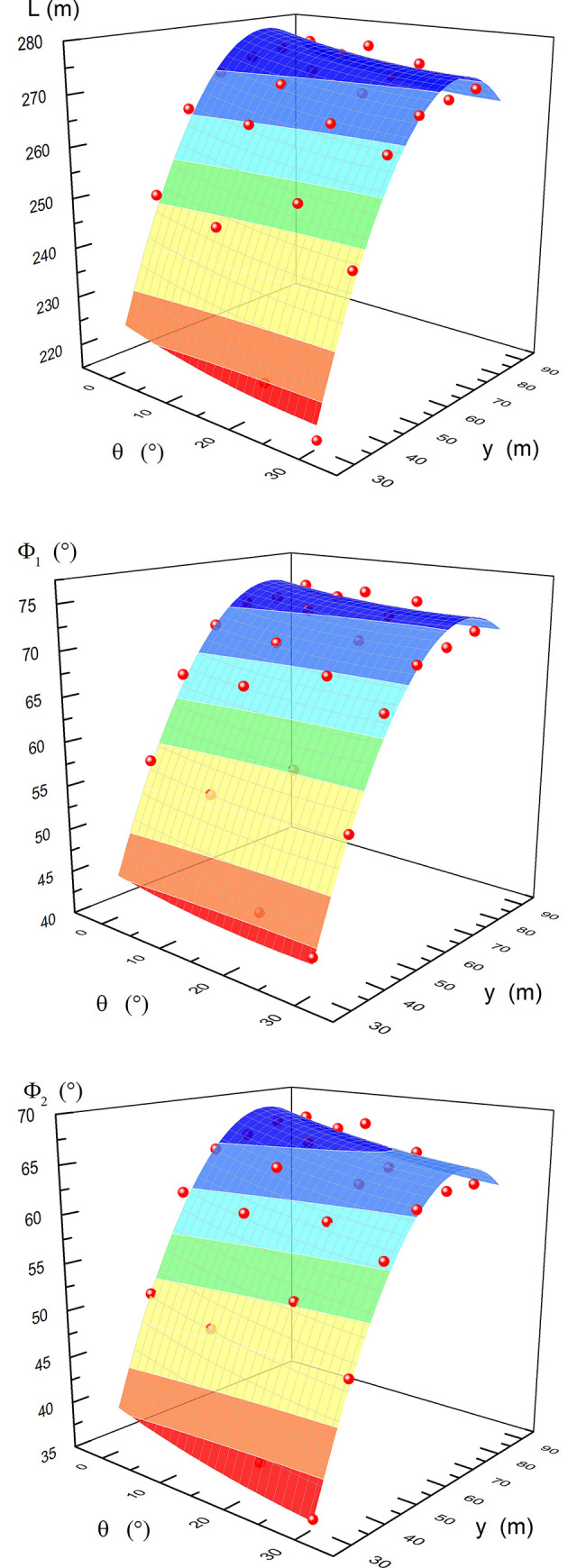
Fitted surfaces. Note: the red balls are the actual values. (A) Pressure relief protection length along strike. (B) Pressure relief angle at the open-off cut side. (C) Pressure relief angle at the mining side.

According to the distribution of the swelling capacity of coal seam #2 in the strike direction, the pressure relief protection parameters under the 24 conditions of the model can be obtained (as shown by the red balls in Fig 10), where *L* is the pressure relief protection length in the strike, m; *Φ*_1_ is the pressure relief angle at the open-off cut side, °; and *Φ*_2_ is the pressure relief angle at the mining side, °.

*θ* and *y* are independent variables, and *L*, *Φ*_1_ and *Φ*_2_ are dependent variables. Using Origin, the Parabola2D fit is selected for the fitting process; the fitting equations are shown in Eqs (6), (7) and (8), respectively, and the fitting diagrams are shown in Fig 10.

$$L=127.9028-0.3442\theta +4.0420y+0.0055\theta^{2}-0.0270y^{2}\;R^{2}=0.9327$$ (6)

$$\Phi_{1}=-5.2632-0.1318\theta +2.0725y+0.0026\theta^{2}-0.0133y^{2}\;R^{2}=0.9666$$ (7)

$$\Phi_{2}=-10.175-0.2124\theta +2.0715y+0.0026\theta^{2}-0.0136y^{2}\;R^{2}=0.9595$$ (8)

*L*, *Φ*_1_ and *Φ*_2_ are mainly affected by *y*; they increase with increasing *y*, and the increasing speed gradually decreases. When y = 70 m, *L*, *Φ*_1_ and *Φ*_2_ tend to be constant. *L*, *Φ*_1_ and *Φ*_2_ are slightly affected by *θ*, and they decrease with increasing *θ*. Under the same conditions, *Φ*_1_>*Φ*_2_, and the pressure relief angle at the open-off cut side (*Φ*_1_) quickly increases to 70° and then stabilizes. As thin coal seams are not easy to mine, the strip width should be as small as possible. According to the simulation results, the optimal strip width is 70 m.

Under the condition of a known coal seam dip, according to fitting Eq (6), the strip width required by the seam pressure relief protection in the strike direction can be obtained. From the obtained strip width, the pressure relief angle can be obtained according to fitting Eqs (7) and (8), that is, the strip position corresponding to the protected seam can be inverted in the strike direction.

The optimal strip width obtained by the numerical simulations is within the range of similarity model experiment results. The law that the pressure relief angle at the open-off cut side is greater than the pressure relief angle at the mining side obtained by the numerical simulations is consistent with the law of the breaking angle at both sides obtained by the similarity model experiment. The pressure relief angle at the open-off cut side obtained by the numerical simulation is 70°, which is greater than the break angle at the open-off cut side obtained by the similarity model experiment, which is 60°. This is because the pressure relief range is greater than the breaking range, and the numerical model is established under ideal conditions. The results of the similarity model experiment verify the results of the numerical simulations.

## Coal seam #2 mining after the double strip mining in the underside protective seam

### The stability of coal pillar during strip mining

Scholars have previously evaluated the stability of coal pillars in strip mining [60–64]. The calculation of the width of the yield zone of the coal pillar is an important part of the stability analysis of the coal pillar. The corresponding empirical formula is as follows [65, 66]:

W=0.00492mH (9)

where *W* is the width of the yield zone, m, *m* is the mining height, m, and *H* is the burial depth, m.

To ensure the long-term stability of a coal pillar, if the coal seam is weak, the ratio should be larger than 0.65 [67, 68]. If the safety factor is larger than 1.6, it can ensure the long-term stability of the coal pillars [69, 70]. The calculation methods of the ratio and the safety factor are shown in Eq (10) and Eq (11) respectively [71, 72].

$$r=\frac{P-2W}{P}$$ 10

$$F=\frac{P_{u}}{P_{a}}$$ (11)

where *r* is the ratio; (*P*-2*W*) is the pillar core width; *P* is the pillar width, m; *F* is the safety factor; *P*_*u*_ is the ultimate coal pillar load; and *P*_*a*_ is the actual coal pillar load.

The expressions of *P*_*u*_ and *P*_*a*_ are shown in Eq (12) and Eq (13) [66].

$$P_{u}=4\gamma H(P-W)$$ (12)

$$P_{a}=\gamma H\left[P+\frac{Y}{2}(2-\frac{Y}{0.6H})\right]$$ (13)

where *γ* is the average volume weight of the overlying strata, kN/m^3^, and *Y* is the mining strip width, m.

To ensure the safety of the coal pillar, the maximum burial depth (600 m) and mining height (1.3 m) are used to calculate the yield zone width according to Eq (9). The yield zone width is approximately 3.84 m. The strength of the coal seam #4 is weak; thus, the ratio (r) should be larger than 0.65, according to Eq (10), and the coal pillar width should be larger than 21.94 m.

When the strip width (*Y*) is 70 m, to maintain a safety factor (*F*) greater than 1.6, according to Eq (11), the pillar width (*P*) needs to be greater than 48.53 m; thus, Y = 70 m, and P = 50 m. According to Eq (10) and Eq (11), *r* = 0.85 and *F* = 1.63, which can ensure the long-term stability of a coal pillar. A method for calculating the coal pillar width based on the strip width is proposed.

### The double-strip numerical model

**Fig 11:**
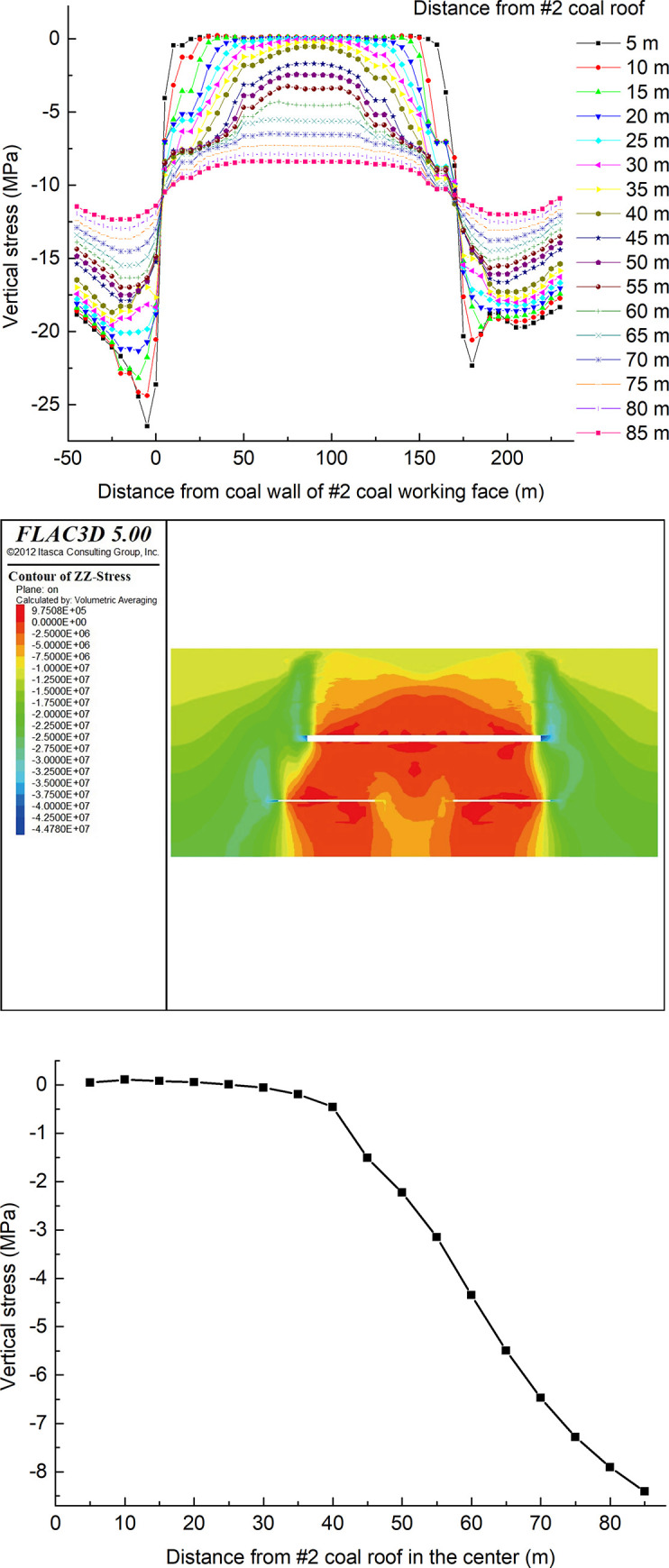
Distribution of vertical stress of the overlying rock of coal seam #2. (A) The dip direction. (B) The dip direction. (C) The vertical direction.

The dip (y-axis direction), strike (x-axis direction), height (z-axis direction) and dip angle of the model are 200 m, 500 m, 250 m, and 0°, respectively. The remaining parameters of the numerical model remain unchanged. The coal seam #2 is mined in a working face layout with a double-strip width of 70 m and a coal pillar width of 50 m, and then the coal seam #2 is mined with a width of 175 m. A row of stress and displacement monitoring points are set at the central position along with the dip at intervals of 5 m, high on the roof of the coal seam #2, and the highest point is 85 m from the roof of the coal seam #2. The vertical stress distribution of the coal seam #2 overburden in the dip direction and the vertical stress distribution at the center point in the vertical direction after the mining 300 m are shown in Fig 11.

After the coal seam #2 is mined, the vertical stress in the goaf is reduced to a pressure relief zone, the minimum stress is reduced to 0 MPa, and the coal walls at both sides show stress concentration. Affected by the strip mining of the coal seam#4, the stress concentration on the left side is greater than that on the right side after coal seam mining #2. This is because the coal seam #4 corresponding to the left side of the underside protective layer is the first mining strip, and the pressure relief is greater there.

Along the height of the roof of the coal seam #2, the vertical stress at the center of the goaf begins to increase when the distance from the roof is 25 m, and the maximum vertical stress difference at the center of the goaf is 60 m from the roof. It can be inferred that the heights of the caving zone and the fractured zone are 25 m and 60 m, respectively.

**Fig 12:**
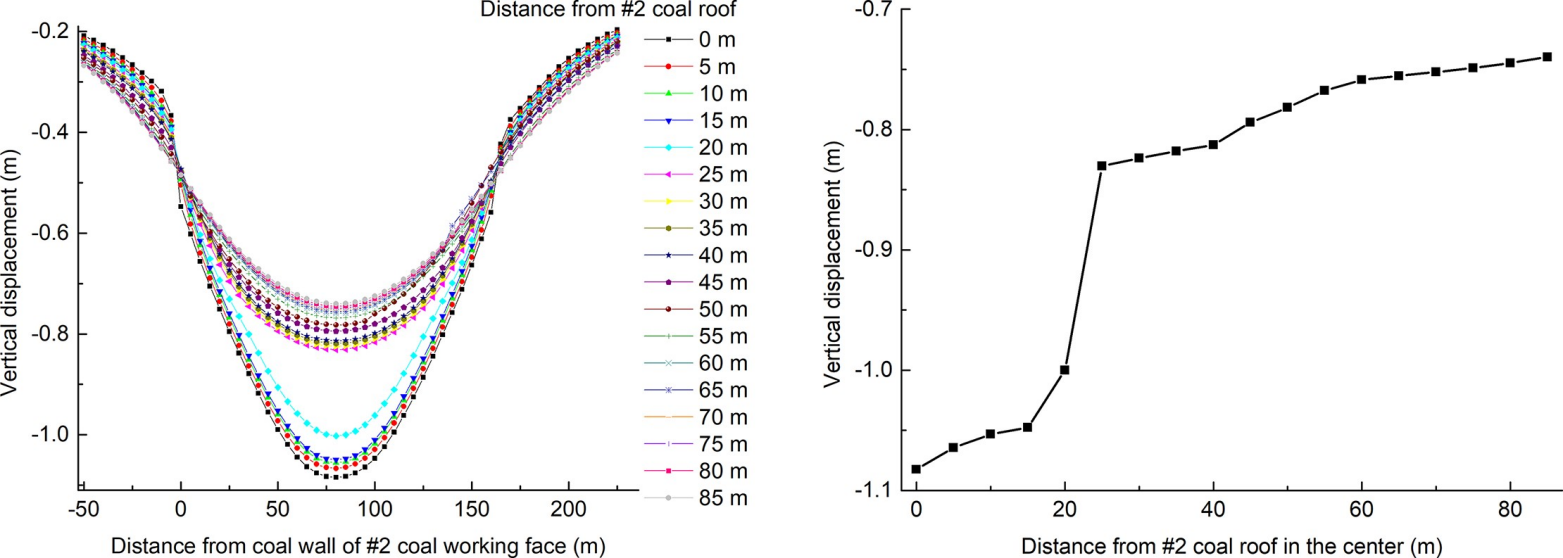
Distribution of vertical displacement in the overlying rock of the coal seam #2. (A) The dip direction. (B) The vertical direction.

The vertical displacement distribution of the overburden of the coal seam #2 in the dip direction and the vertical displacement distribution at the center point in the vertical direction are shown in Fig 12.

The vertical displacement change law is the same as the vertical stress change law. Along with the height of the roof of coal seam #2, when the distance from the roof is 20~25 m, the vertical displacement gradient of the goaf center point is the largest, and when the distance from the roof is 60 m, the rate of increase in the vertical displacement of the goaf center point decreases. It can be inferred that the heights of the caving zone and the fractured zone are 25 m and 60 m, respectively.

The height of the caving zone obtained by numerical simulation of the coal seam #2 overlying strata is greater than that of the theoretical calculation. The height of the fracture zone obtained by numerical simulation is consistent with that of the theoretical calculation. The underside protective seam mining increased the development height of the caving zone of the protected seam, indicating that the double-strip mining of coal seam #4 had a pressure relief protection effect on coal seam #2. The rationality of the designed protective seam strip width and coal pillar width is verified.

## Conclusions

1) Through the changes in strain during underside protective coal seam #4 mining at a similarity model experiment, it was concluded that the optimal range of the strip width of the coal seam #4 is 55~90 m. The coal seam #2 mining verified the pressure relief protection effect.

2) Through the numerical simulation of protective seam mining under different dip angles and strip widths, it is concluded that *Z* is greatly affected by *y*, *Z* has an approximately linear relationship with *y*, and *Z* reaches its maximum value at *θ* = 16.7°; *Γ*_1_ and *Γ*_2_ are mainly affected by *θ*. *L*, *Φ*_1_ and *Φ*_2_ are mainly affected by *y*, they increase with increasing *y*, and the rate of increase speed gradually decreases. The dip angle of the coal seam for the best pressure relief effect is 16.7°, and the corresponding strip width is 70 m. According to the fitting equation, the strip width and the strip position corresponding to the protected seam required for the protected seam width in the dip and strike can be obtained by inversion.

3) The results of the similarity model experiment verify the results of numerical simulations. The optimal strip width obtained by the numerical simulations is within the range of similarity model experiment results. The results of the numerical simulations suggest that the pressure relief angle at the open-off cut side is greater than the pressure relief angle at the mining side, consistent with the law of the breaking angle at both sides obtained by the similarity model experiment.

4) A method for calculating the coal pillar width based on the strip width is proposed. According to the related theory of the stability of coal pillars in strip mining, a strip width of 70 m and pillar width of 50 m can ensure the long-term stability of a coal pillar.

5) According to the laws of the vertical stress and vertical displacement of the overlying strata based on double-strip numerical simulation, it is concluded that the height of the caving zone is 25 m greater than that of the theoretical calculation and that the height of the fracture zone is 60 m, consistent with the results of the theoretical calculation. The underside protective seam mining increased the development height of the caving zone of the protected seam and verified the pressure relief protection effect of the designed protective seam strip width and pillar width.

## Supporting information

S1 File (PDF)

## References

[1] Balat H. Role of Coal in Sustainable Energy Development. Energy Exploration & Exploitation. 2016;25(3):151–74. 10.1260/014459807782009169.

[2] Gawlik L, Kaliski M, Kamiński J, Sikora AP, Szurlej A. Hard Coal in the Fuel-Mix Of Poland: The Long-Term Perspective. Archives of Mining Sciences. 2016;61(2):335–50. 10.1515/amsc-2016-0025.

[3] Kasap Y, Şensöğüt C, Ören Ö. Efficiency change of coal used for energy production in Turkey. Resources Policy. 2020;65:101577 10.1016/j.resourpol.2019.101577.

[4] Madhavi M, Nuttall WJ. Coal in the twenty-first century: a climate of change and uncertainty. Proceedings of the Institution of Civil Engineers—Energy. 2019;172(2):46–63. 10.1680/jener.18.00011.

[5] Sovacool BK, Cooper C, Parenteau P. From a hard place to a rock: Questioning the energy security of a coal-based economy. Energy Policy. 2011;39(8):4664–70. 10.1016/j.enpol.2011.04.065.

[6] Wang N, Shen R, Wen Z, De Clercq D. Life cycle energy efficiency evaluation for coal development and utilization. Energy. 2019;179:1–11. 10.1016/j.energy.2019.04.111.

[7] Wyn HK, Konarova M, Beltramini J, Perkins G, Yermán L. Self-sustaining smouldering combustion of waste: A review on applications, key parameters and potential resource recovery. Fuel Processing Technology. 2020;205:106425 10.1016/j.fuproc.2020.106425.

[8] Yılmaz AO, Aydiner K. The Place of Hard Coal in Energy Supply Pattern of Turkey. Energy Sources, Part B: Economics, Planning, and Policy. 2009;4(2):179–89. 10.1080/15567240701620457.

[9] Çelik PA, Aksoy DÖ, Koca S, Koca H, Çabuk A. The approach of biodesulfurization for clean coal technologies: a review. International Journal of Environmental Science and Technology. 2019;16(4):2115–32. 10.1007/s13762-019-02232-7.

[10] Crowe JA, Li R. Is the just transition socially accepted? Energy history, place, and support for coal and solar in Illinois, Texas, and Vermont. Energy Research & Social Science. 2020;59:101309 10.1016/j.erss.2019.101309.

[11] Khoonthiwong C, Saengkaew P, Chankow N. Determination of the ash content of coal samples by nuclear techniques with bismuth germanate detectors. International Journal of Coal Preparation and Utilization. 2020;2020:1–10. 10.1080/19392699.2020.1729137. WOS:000514282000001.

[12] Konovsek D, Praunseis Z, Avsec J, Bercic G, Pohar A, Zavsek S, et al Underground coal gasification—the Velenje Coal Mine energy and economic calculations. Chemical Industry and Chemical Engineering Quarterly. 2017;23(2):269–77. 10.2298/CICEQ160504042K.

[13] Leonard R, Zulfikar R, Stansbury R. Coal mining and lung disease in the 21st century. Curr Opin Pulm Med. 2020;26(2):135–41. 10.1097/MCP.0000000000000653 . 31815751

[14] Liu A, Liu S, Wang G, Sang G. Modeling of Coal Matrix Apparent Strains for Sorbing Gases Using a Transversely Isotropic Approach. Rock Mechanics and Rock Engineering. 2020;53(9):4163–81. 10.1007/s00603-020-02159-3.

[15] Liu S, Li X, Wang D, Zhang D. Experimental Study on Temperature Response of Different Ranks of Coal to Liquid Nitrogen Soaking. Natural Resources Research. 2020 10.1007/s11053-020-09768-3.

[16] Karacan CÖ, Esterhuizen GS, Schatzel SJ, Diamond WP. Reservoir simulation-based modeling for characterizing longwall methane emissions and gob gas venthole production. International Journal of Coal Geology. 2007;71(2–3):225–45. 10.1016/j.coal.2006.08.003.

[17] Mondal D, Roy PNS, Kumar M. Monitoring the strata behavior in the Destressed Zone of a shallow Indian longwall panel with hard sandstone cover using Mine-Microseismicity and Borehole Televiewer data. Engineering Geology. 2020;271:105593 10.1016/j.enggeo.2020.105593.

[18] Palchik V. Formation of fractured zones in overburden due to longwall mining. Environmental Geology. 2003;44(1):28–38. 10.1007/s00254-002-0732-7.

[19] Qu Q, Xu J, Wu R, Qin W, Hu G. Three-zone characterisation of coupled strata and gas behaviour in multi-seam mining. Int J Rock Mech Min. 2015;78:91–8. 10.1016/j.ijrmms.2015.04.018.

[20] Singh MM, Kendorski FS. Strata disturbance prediction for mining beneath surface water and waste impoundments. International Journal of Rock Mechanics and Mining Sciences & Geomechanics Abstracts. 1983;20(1):A13 10.1016/0148-9062(83)91724-2.

[21] Han Y, Cheng J, Huang Q, Zou DHS, Zhou J, Huang S, et al Prediction of the height of overburden fractured zone in deep coal mining: case study. Archives of Mining Sciences. 2018;63(3):617–31. 10.24425/123687.

[22] Suchowerska AM, Carter JP, Merifield RS. Horizontal stress under supercritical longwall panels. Int J Rock Mech Min. 2014;70:240–51. 10.1016/j.ijrmms.2014.03.009.

[23] Sun Y, Zuo J, Karakus M, Wang J. Investigation of movement and damage of integral overburden during shallow coal seam mining. Int J Rock Mech Min. 2019;117:63–75. 10.1016/j.ijrmms.2019.03.019.

[24] Wang H, Zhang D, Wang X, Zhang W. Visual Exploration of the Spatiotemporal Evolution Law of Overburden Failure and Mining-Induced Fractures: A Case Study of the Wangjialing Coal Mine in China. Minerals. 2017;7(3):35 10.3390/min7030035.

[25] Wen J, Cheng W, Chen L, Shi S, Wen Z. A study of the dynamic movement rule of overlying strata combinations using a short‐wall continuous mining and full‐caving method. Energy Science & Engineering. 2019;7(6):2984–3004. 10.1002/ese3.474.

[26] Zhou Y, Li M, Xu X, Li M. A study on dual-load-zone model of overlying strata and evolution law of mining stress. Computers, Materials & Continua. 2019;58(2):391–407. 10.32604/cmc.2019.04456.

[27] Liang Y, Li B, Zou Q. Movement type of the first subordinate key stratum and its influence on strata behavior in the fully mechanized face with large mining height. Arabian Journal of Geosciences. 2019;12(2). 10.1007/s12517-018-4208-9.

[28] Gao Y, Gao F, Yeung MR. Modeling large displacement of rock block and a work face excavation of a coal mine based on discontinuous deformation analysis and finite deformation theory. Tunnelling and Underground Space Technology. 2019;92:103048 10.1016/j.tust.2019.103048.

[29] Sampath KHSM, Perera MSA, Elsworth D, Ranjith PG, Matthai SK, Rathnaweera T, et al Effect of coal maturity on CO2-based hydraulic fracturing process in coal seam gas reservoirs. Fuel. 2019;236:179–89. 10.1016/j.fuel.2018.08.150.

[30] Xie J, Xu J. Effect of key stratum on the mining abutment pressure of a coal seam. Geosciences Journal. 2017;21(2):267–76. 10.1007/s12303-016-0044-7.

[31] Adhikary DP, Guo H. Modelling of Longwall Mining-Induced Strata Permeability Change. Rock Mechanics and Rock Engineering. 2014;48(1):345–59. 10.1007/s00603-014-0551-7.

[32] Cheng G, Ma T, Tang C, Liu H, Wang S. A zoning model for coal mining—induced strata movement based on microseismic monitoring. Int J Rock Mech Min. 2017;94:123–38. 10.1016/j.ijrmms.2017.03.001.

[33] Ghabraie B, Ren G, Smith J, Holden L. Application of 3D laser scanner, optical transducers and digital image processing techniques in physical modelling of mining-related strata movement. Int J Rock Mech Min. 2015;80:219–30. 10.1016/j.ijrmms.2015.09.025.

[34] Li J, Huang Y, Zhang J, Li M, Qiao M, Wang F. The influences of key strata compound breakage on the overlying strata movement and strata pressure behavior in fully mechanized caving mining of shallow and extremely thick seams: a case study. Advances in Civil Engineering. 2019;2019:1–11. 10.1155/2019/5929635.

[35] Yang D, Guo W, Tan Y. Study on the evolution characteristics of two-zone failure mode of the overburden strata under shallow buried thick seam mining. Advances in Civil Engineering. 2019;2019:1–9. 10.1155/2019/9874769.

[36] Ghabraie B, Ren G, Smith JV. Characterising the multi-seam subsidence due to varying mining configuration, insights from physical modelling. Int J Rock Mech Min. 2017;93:269–79. 10.1016/j.ijrmms.2017.02.001.

[37] Wang G, Wu M, Wang R, Xu H, Song X. Height of the mining-induced fractured zone above a coal face. Engineering Geology. 2017;216:140–52. 10.1016/j.enggeo.2016.11.024.

[38] He C, Xu J, Wang F, Wang F. Movement boundary shape of overburden strata and Its influencing factors. Energies. 2018;11(4):742 10.3390/en11040742.

[39] Kang H, Lou J, Gao F, Yang J, Li J. A physical and numerical investigation of sudden massive roof collapseduring longwall coal retreat mining. International Journal of Coal Geology. 2018;188:25–36. 10.1016/j.coal.2018.01.013.

[40] Le TD, Oh J, Hebblewhite B, Zhang C, Mitra R. A discontinuum modelling approach for investigation of Longwall Top Coal Caving mechanisms. Int J Rock Mech Min. 2018;106:84–95. 10.1016/j.ijrmms.2018.04.025.

[41] Yang W, Xia X. Study on mining failure law of the weak and weathered composite roof in a thin bedrock working face. Journal of Geophysics and Engineering. 2018;15(6):2370–7. 10.1088/1742-2140/aacedf.

[42] Chai J, Du W. Experimental study on the application of BOTDA in the overlying strata deformation monitoring induced by coal mining. Journal of Sensors. 2019;2019:1–9. 10.1155/2019/3439723.

[43] Pan W, Nie X, Li X. Effect of premining on hard roof distress behavior: a case study. Rock Mechanics and Rock Engineering. 2019;52(6):1871–85. 10.1007/s00603-018-1657-0.

[44] Zhou D, Wu K, Bai Z, Hu Z, Li L, Xu Y, et al Formation and development mechanism of ground crack caused by coal mining: effects of overlying key strata. Bulletin of Engineering Geology and the Environment. 2019;78(2):1025–44. 10.1007/s10064-017-1108-2.

[45] Li H, Guo G, Zhai S. Mining scheme design for super-high water backfill strip mining under buildings: a Chinese case study. Environmental Earth Sciences. 2016;75(12). 10.1007/s12665-016-5837-5.

[46] Gao R, Yu B, Xia H, Duan H. Reduction of Stress Acting on a Thick, Deep Coal Seam by Protective-Seam Mining. Energies. 2017;10(8):1209 10.3390/en10081209.

[47] Guowei D, Yinhui Z. A Novel Method for Selecting Protective Seam against Coal and Gas Outburst: A Case Study of Wangjiazhai Coal Mine in China. Sustainability. 2017;9(6):1015 10.3390/su9061015.

[48] Zhang C, Yu L, Feng R, Zhang Y, Zhang G. A Numerical Study of Stress Distribution and Fracture Development above a Protective Coal Seam in Longwall Mining. Processes. 2018;6(9):146 10.3390/pr6090146.

[49] Tu Q, Cheng Y. Stress evolution and coal seam deformation through the mining of a remote upper protective layer. Energy Sources, Part A: Recovery, Utilization, and Environmental Effects. 2018:1–11. 10.1080/15567036.2018.1518352.

[50] Jia H, Wang K, Xu C, Fu Q. Permeability distribution characteristics of underlying coal seam disturbed by mining activity. Energy Sources, Part A: Recovery, Utilization, and Environmental Effects. 2019:1–16. 10.1080/15567036.2019.1657207.

[51] Fang F, Shu C, Wang H. Physical simulation of upper protective coal layer mining with different coal seam inclinations. Energy Science & Engineering. 2020 10.1002/ese3.740.

[52] Zhang Y. Distribution law of floor stress during mining of the upper protective coal seam. Sci Prog. 2020;103(3):36850420930982 10.1177/0036850420930982 . 32579431 PMC10451935

[53] Guo C, Yang Z, Li S, Lou J. Predicting the Water-Conducting Fracture Zone (WCFZ) Height Using an MPGA-SVR Approach. Sustainability. 2020;12(5):1809 10.3390/su12051809.

[54] Yavuz H. An estimation method for cover pressure re-establishment distance and pressure distribution in the goaf of longwall coal mines. Int J Rock Mech Min. 2004;41(2):193–205. 10.1016/S1365-1609(03)00082-0.

[55] Zhang K, Yang T, Bai H, Pathegama Gamage R. Longwall mining–induced damage and fractures: field measurements and simulation using FDM and DEM coupled method. International Journal of Geomechanics. 2018;18(1):04017127 10.1061/(asce)gm.1943-5622.0001040.

[56] Lu W, He C, Zhang X. Height of overburden fracture based on key strata theory in longwall face. PLoS One. 2020;15(1):e0228264 10.1371/journal.pone.0228264 31978195 PMC6980533

[57] Wu Q, Shen J, Liu W, Wang Y. A RBFNN-based method for the prediction of the developed height of a water-conductive fractured zone for fully mechanized mining with sublevel caving. Arabian Journal of Geosciences. 2017;10(7). 10.1007/s12517-017-2959-3.

[58] Li H. Similar simulation test of mine pressure Xuzhou: China University of Mining and Technology Press; 1988.

[59] Zhu H, Fang S, Huo Y, Guo J, Wu Y, Hu L. Study of the Dynamic Development Law of Overburden Breakage on Mining Faces. Sci Rep. 2020;10(1):6555 10.1038/s41598-020-63526-2 32300230 PMC7162937

[60] Chen S, Qu X, Yin D, Liu X, Ma H, Wang aH. Investigation Lateral Deformation and Failure Characteristics of Strip Coal Pillar in Deep Mining. Geomech Eng. 2018;14(5):421–8. 10.12989/gae.2018.14.5.421.

[61] Liu S, Wan Z, Zhang Y, Lu S, Ta X, Wu Z. Research on evaluation and control technology of coal pillar stability based on the fracture digitization method. Measurement. 2020;158:107713 10.1016/j.measurement.2020.107713.

[62] Sun W, Zhang Q, Luan Y, Zhang X-P. A study of surface subsidence and coal pillar safety for strip mining in a deep mine. Environmental Earth Sciences. 2018;77(17). 10.1007/s12665-018-7810-y.

[63] Wang B, Dang F, Gu S, Huang R, Miao Y, Chao W. Method for determining the width of protective coal pillar in the pre-driven longwall recovery room considering main roof failure form. Int J Rock Mech Min. 2020;130:104340 10.1016/j.ijrmms.2020.104340.

[64] Wang R, Bai J-b, Yan S, Chang Z-g, Wang X-y. An innovative approach to theoretical analysis of partitioned width & stability of strip pillar in strip mining. Int J Rock Mech Min. 2020;129:104301 10.1016/j.ijrmms.2020.104301.

[65] Gao W, Ge M. Stability of a coal pillar for strip mining based on an elastic-plastic analysis. Int J Rock Mech Min. 2016;87:23–8. 10.1016/j.ijrmms.2016.05.009.

[66] Wilson AH, DP A. Research into the determination of pillar size. Mining Engineer. 1972;31:409–30.

[67] Gao W. Elastic-Plastic Mechanics Analysis on Stability of Coal Pillar. Advanced Materials Research. 2008;33–37:1123–8. 10.4028/www.scientific.net/AMR.33-37.1123.

[68] Sheorey PR, Das MN, Bordia SK, Singh B. Pillar strength approaches based on a new failure criterion for coal seams. International Journal of Mining and Geological Engineering. 1986;4(4):273–90. 10.1007/bf01552957.

[69] Jawed M. Chronological development in coal pillar design for bord and pillar workings: A critical appraisal. Journal of Geology and Mining Research. 2013;5(1):1–11. 10.5897/JGMR12.010.

[70] Loui JP, Sheorey PR. Estimation of non-effective width for different panel shapes in room and pillar extraction. Int J Rock Mech Min. 2002;39(1):95–9. 10.1016/S1365-1609(01)00074-0.

[71] Guo Q, Guo G, Lv X, Zhang W, Lin Y, Qin S. Strata movement and surface subsidence prediction model of dense solid backfilling mining. Environmental Earth Sciences. 2016;75(21). 10.1007/s12665-016-6237-6.

[72] Guo W, Wang H, Chen S. Coal pillar safety and surface deformation characteristics of wide strip pillar mining in deep mine. Arabian Journal of Geosciences. 2016;9(2). 10.1007/s12517-015-2233-5.

